# Nizatidine Improves Impaired Salivary Secretion in GERD

**DOI:** 10.4137/ccrep.s870

**Published:** 2008-08-19

**Authors:** Yoshihisa Urita, Toshiyasu Watanabe, Kazuo Hike, Makie Nanami, Tadashi Maeda, Yosuke Sasaki, Asuka Nakayama, Takamasa Ishii, Kaoru Domon, Susumu Ishihara, Masaki Sanaka, Hitoshi Nakajima, Motonobu Sugimoto

**Affiliations:** From the Department of General Medicine and Emergency Care, Toho University, Tokyo, Japan. Division of Gastroenterology and Hepatology, Toho University, Tokyo, Japan. Department of Hematology, Toho University, Tokyo, Japan.

**Keywords:** salivary scintigraphy, GERD, nizatidine, laryngeal discomfort

## Abstract

During esophageal acid clearance, salivation plays an important role in defending the esophageal mucosa. Nizatidine, a histamine H2 receptor antagonist, inhibits acetylcholine esterase, with a resultant increase in acetylcholine. We experienced a patient with gastroesophageal reflux disease (GERD) and impaired salivary secretion who has been successfully treated with nizatidine.

**Case report:**

A 63-year-old female visited our hospital with complaints of heartburn and continuous laryngeal discomfort. Saliva scintigraphy was performed to evaluate the salivary function. Washout ratio was decreased to be 25%–40% in individual salivary gland. After the treatment with nizatidine, salivary scintigraphy demonstrated the increased washout ratios. The values of both parotid glands increased up to 90%, whereas those of submandibular glands improved to be around a normal range. GERD symptoms disappeared completely after treatment. In conclusion, nizatidine may be one of therapeutic options for low salivary excretion.

## Introduction

Symptoms of gastro-esophageal reflux disease (GERD) are common, affecting 10%–30% of the population in Western countries [1]. Recently, the prevalence of GERD is also increasing in a Japanese population with a high prevalence of atrophic gastritis [2]. GERD symptoms can be divided into typical symptoms (heartburn and acid regurgitation) and atypical symptoms. Heartburn and acid regurgitation are more prevalent in clinical practice and their correlation with GERD has been established. However, it may be difficult to establish whether some atypical symptoms such as laryngeal symptoms are due to reflux in the individual patient. The major abnormalities associated with the development of GERD are related to incompetence of the antireflux barrier and impairment of esophageal luminal clearance after reflux [3, 4]. During esophageal acid clearance, salivation plays an important role in defending the esophageal mucosa [5, 6]. Nizatidine, a histamine H2 receptor antagonist, inhibits acetylcholine esterase, with a resultant increase in acetylcholine [7]. In healthy volunteers, increased salivary secretion has been induced by nizatidine. However, it has been unclear whether nizatidine improve the low salivary secretion in GERD patients. We experienced a GERD patient with impaired salivary secretion who has been successfully treated with nizatidine.

## Case Report

**Figure 1:**
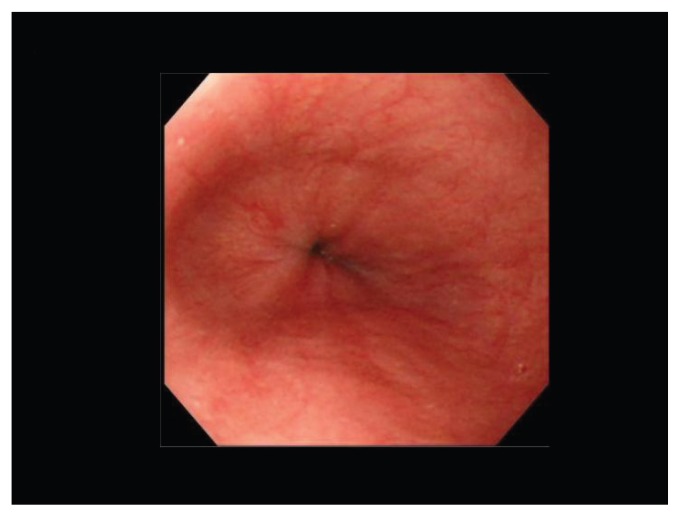
An upper endoscopic examination reveals that there were no abnormal findings of the esophagus, including a mucosal break, hiatal hernia, and whitish mucosa.

A 63-year-old female visited Toho University Omori Hospital with complaints of heartburn and persistent laryngeal discomfort. She received an endoscopic examination two years ago as a further examination of gastric cancer screening. At that time, atrophic gastritis was pointed out and Helicobacter pylori (H.pylori) infection was positive. Although H.pylori was successfully treated with a PPI-based triple therapy, laryngeal discomfort had not been disappeared. First, an upper endoscopic examination was performed, but abnormal findings of the esophagus, including a mucosal break, hiatal hernia, and whitish mucosa, were not detected (Fig. 1). Therefore, saliva scintigraphy was performed to evaluate the salivary function. In our previous study [8], we defined the optimal cutoff point for determining the decreased salivary secretion as 51% in parotid glands and 36% in submandibular glands.

After an overnight fasting, saliva scintigraphy was performed with the patient in the supine position under a gamma camera with high-resolution collimators. No oral stimulus was permitted before and during imaging. Following intravenous injection of 180 to 200 Mbq 99 mTc-pertechnetate, anterior sequential imaging was performed every minute for 40 minutes. At 20 minutes after injection of radio-nuclide, a lemon candy was administrated intraorally to stimulate salivary secretion. Regions of Interests (ROI) were selected on the individual submandibular and parotid glands, oral cavity, and thyroid gland. Time activity curves were drawn for each of these. Washout ratio (peak count before lemon candy administration-lowest count after administration/peak count before administration) was examined.

**Figure 2:**
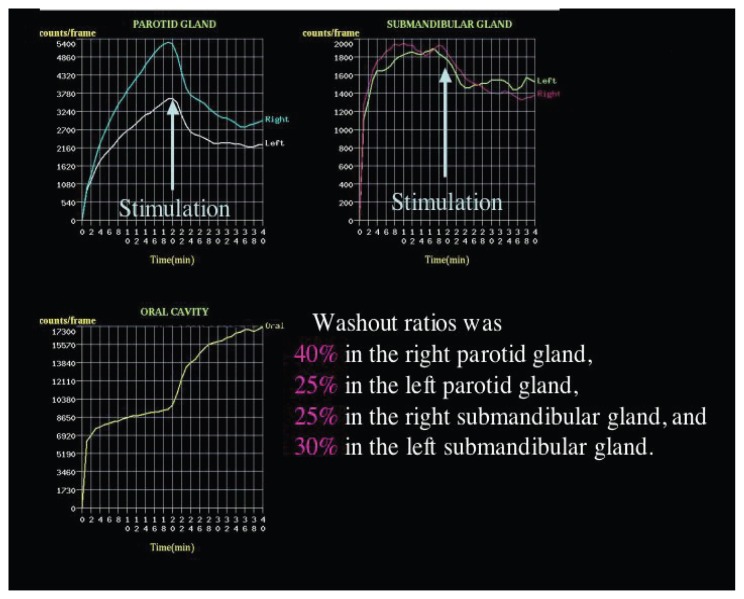
Saliva scintigraphy was performed to evaluate the salivary function before treatment with nizatidine. Washout ratio was decreased in the parotid glands.

Washout ratio was 40% in the right parotid gland, 25% in the left parotid gland, 25% in the right submandibular gland, and 30% in the left submandibular gland (Fig. 1). After the first scintigraphy was performed, the patient received 300 mg of nizatidine per day for 2 months based on the treatment for peptic ulcer in Japan. During the two months course of the nizatidine treatment, the patients has not taken any other drugs and there have no possible confounding factors that would also change salivary flow. After the treatment, salivary scintigraphy was done and demonstrated the increased washout ratios in all four major glands (Fig. 2). The value of right parotid gland increased from 40% to 80% and that of left one did from 25% to 78% after treatment with nizatidine. Likewise, the washout ratio of right submandibular gland increased from 25% to 45% and that of left one did from 30% to 51%. GERD symptoms, including heartburn and laryngeal discomfort disappeared completely after treatment. The patient has been followed up for 10 months after nizatidine treatment and GERD symptoms have not reappeared.

## Discussion

GERD refers to the abnormal exposure of the esophageal mucosa to gastric contents. Although GERD symptoms affect 10%–30% of the population in Western countries [1], endoscopic esophagitis is less prevalent, and is reported to occur in up to 2% of individuals [9–10]. Only one-third of GERD patients have endoscopic positive findings, while others have no obvious mucosal breaks even though GERD symptoms are present [11]. The present case has also had a persistent unusual sensation in her throat for a long time although erosive esophagitis is not found endoscopically. Since she presents with mild symptoms, she sought consultation with a general practitioner, not with a gastroenterologist. Nandurkar et al. [12] reported that only about half of patients with GERD symptoms in a community seek health care over a 10-year period and only 19% of them had an endoscopy. Isolauri et al. [13] described that medication was used by only 16% of subjects with symptoms in Norway. The general practitioner should decide whether diagnosis and therapy will be based on symptoms analysis alone or whether further examinations will be undertaken, although it is still difficult for the general practitioner to establish with certainty if the symptoms are truly directly related to refluxed material from the upper digestive tract.

GERD is mainly due to a combination of an increased number of gastroesophageal reflux events with an abnormally prolonged clearance of the refluxed material. Esophageal acid clearance mainly depends on esophageal perstalsis and gravity leaving only a minimal residue that sustains an acidic pH in the esophageal mucosa until it is neutralized by swallowed saliva [14, 15]. Salivary flow, volume, clearance, and alterations in the salivary electrolytic composition can influence the protective capacity of the regional mucous membrane [16, 17]. Other studies have shown that physical and chemical stimuli to the esophagus interfere with the salivary production [6, 16, 18]. Recently, it has been suggested that the lower incidence of GERD in African-American could be correlated with significantly higher levels of salivary mucin [19]. Furthermore, Fraser has shown the close association between GERD and laryngeal symptoms [20]. These previous reports suggest the possible association between salivary disorders and developing laryngeal symptoms. Chronic salivary dysfunction is clinically significant because it may lead to rampant dental destruction, mucosal infection and a variety of speech and digestive disturbances, and in itself may seriously impair the patient’s quality of life [21, 22]. Since the present case has been feeling an unusual sensation in her throat for a longtime even after H.pylori eradication therapy, salivary dysfunction is suspected. Salivary scintigraphy reveals lower wash out rates of four major salivary glands after stimulation, suggesting that the treatment for salivary disorders may improve laryngeal symptoms. Therefore we prescribe nizatidine, a histamine H2 receptor antagonist. The values of both parotid glands increased up to 90%, whereas those of submandibular glands improved to be around a normal range after treatment with nizatidine for two months.

Nizatidine has been reported to inhibit acetylcholine esterase, with a resultant increase in acetylcholine, in the cholinergic system [7]. Adachi et al. [23] reported increased salivary secretion and bicarbonate output by nizatidine. They collected saliva at two hours after ingestion of nizatidine by asking each patient to spit into a collection tube. This method, in which saliva is collected for two hours, seems cumbersome for the patient and bothering the investigator. In contrast, the radioisotopic approach for the assessment of salivary gland function using 99 mTcO^4−^ scintigraphy has been shown noninvasive and practical [24]. Universally this scintigraphy has been used to quantify the uptake and the secretion in individual salivary glands. After treatment with nizatidine, the washout rates of parotid glands increased more greatly, compared to those of submandibular glands. The parotid gland predominantly secretes a protein rich saliva which includes enzymes like amylase while the submandibular secretions are mucin rich which are useful in lubricating the bolus of food [25]. It has been also reported that submandibular glands showed a greater tendency towards profuse unstimulated secretions [26]. Although the mechanisms in which nizatidine improve the salivary secretion of parotid glands more greatly has been unknown, the drug can provoke alterations in saliva composition in oral cavity. As reported Costa et al. [27], reduced volume of saliva may correlate with some of laryngeal symptoms. In the present case, persistent laryngeal symptoms disappeared dramatically when washout rates of salivary glands increased remarkably after treatment.

In conclusion, the GERD patient with impaired salivary function was successfully treated with nizatidine. This suggests that nizatidine may be one of therapeutic options for low salivary excretion although patients with GERD are treated mainly with proton pump inhibitors.

## Figures and Tables

**Figure 3:**
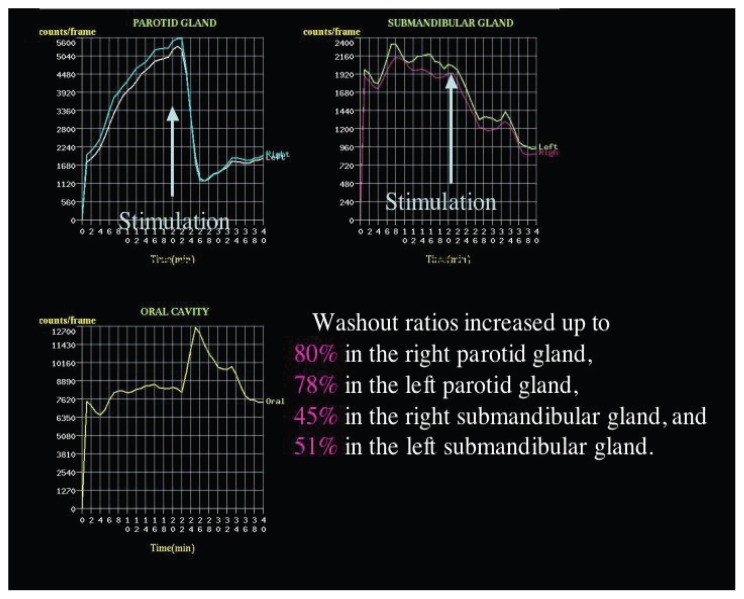
Saliva scintigraphy after treatment with nizatidine demonstrated the increased washout ratios in all four major glands.
